# Screening for lysosomal diseases in a selected pediatric population: the case of Gaucher disease and acid sphingomyelinase deficiency

**DOI:** 10.1186/s13023-023-02797-0

**Published:** 2023-07-21

**Authors:** Maja Di Rocco, Carlo Dionisi Vici, Alberto Burlina, Francesco Venturelli, Agata Fiumara, Simona Fecarotta, Maria Alice Donati, Marco Spada, Daniela Concolino, Andrea Pession

**Affiliations:** 1 Department of Pediatrics, Unit of Rare Diseases IRCCS Istituto Giannina Gaslini, Genoa, Italy; 2 Bambino Gesù Children’Hospital, Rome, Italy; 3 Division of Inherited Metabolic Diseases, Department of Diagnostic Services, University Hospital, Padua, Italy; 4 Pediatric Unit, Istituti di Ricovero e Cura a Carattere Scientifico Azienda Ospedaliero- Universitaria di Bologna, University of Bologna, Bologna, Italy; 5 Referral Center for Inherited Metabolic Disorders, Pediatric Clinical, University-Hospital “Gaspare Rodolico - San Marco”, Catania, Italy; 6 Clinical and Experimental Medicine Department, University of Catania, Catania, Italy; 7 Federico II University, Naples, Italy; 8 Metabolic and Neuromuscular Unit, Meyer Hospital, Florence, Italy; 9 Department of Pediatrics, University of Torino, Torino, Italy; 10 Department of Science of Health, Pediatric Unit, Magna Graecia University of Catanzaro, Catanzaro, Italy

**Keywords:** Selected population screening, Lysosomal Storage Diseases, Gaucher Disease, Acid Sphingomyelinase Deficiency, Splenomegaly

## Abstract

**Background:**

GD and ASMD are lysosomal storage disorders that enter into differential diagnosis due to the possible overlap in their clinical manifestations. The availability of safe and effective enzymatic therapies has recently led many investigators to develop and validate new screening tools, such as algorithms, for the diagnosis of LSDs where the lack of disease awareness or failure to implement newborn screening results in a delayed diagnosis.

**Results:**

the proposed algorithm allows for the clinical and biochemical differentiation between GD and ASMD. It is based on enzyme activity assessed on dried blood spots by multiplexed tandem mass spectrometry (MS/MS) coupled to specific biomarkers as second-tier analysis.

**Conclusions:**

we believe that this method will provide a simple, convenient and sensitive tool for the screening of a selected population that can be used by pediatricians and other specialists (such as pediatric hematologists and pediatric hepatologists) often engaged in diagnosing these disorders.

## Background

Lysosomal Storage Diseases (LSDs) are a family of genetic diseases characterized by the pathologic accumulation of sphingolipids in lysosomes, leading to multisystemic and progressive organ dysfunction.

Gaucher disease (GD) is one of the most prevalent among all LSDs; the deficient activity of the enzyme glucocerebrosidase leads to the accumulation of glucocerebroside in reticuloendothelial cells (Gaucher cells), which accumulate in various organs and trigger an inflammatory response resulting in a wide spectrum of clinical presentations. The main recognized subtype of GD are (a) Type I GD (chronic visceral non-neuronopathic form), (b) Type II GD (infantile acute neuronopathic form), a rapidly progressive neurovisceral storage disease, which leads to death in the first years of life, and (c) Type III GD (chronic neuronopathic form), a slowly progressive neurovisceral form. Type I GD is the most frequent form. It is mainly characterized by splenomegaly, hepatomegaly, thrombocytopenia, anemia, and various bone manifestations, with an onset ranging from childhood/adolescence to adulthood. However, the phenotypic expression and the clinical course of the disease are extremely heterogeneous, with significant disease severity and organ involvement variation among individuals diagnosed with the same GD subtype. Enzyme replacement therapy (ERT) is available since the early 1990s for the treatment of GD type I and III, but not for GD type II since ERT does not cross the blood-brain barrier. Furthermore, the more recent oral substrate reduction therapy (SRT) is approved only for GD type I, no other therapy is available for type II GD due to the rapid neurodegeneration [1, 2].

Acid Sphingomyelinase Deficiency (ASMD), formerly known as Niemann Pick disease type A and B, is an autosomal recessive LSD caused by the deficiency of the enzyme acid sphingomyelinase, which leads to sphingomyelin accumulation with a wide spectrum of clinical presentations. Based on the degree of enzyme deficiency, three main subtypes are recognized: (a) Type A (infantile acute neurovisceral form), characterized by rapidly progressive neurodegeneration and death in the first years of life (b) Type B (chronic visceral non-neuronopathic form) and (c) Type A-B (intermediate chronic neurovisceral form) [3, 4]. Type B ASMD can be characterized by splenomegaly, hepatomegaly, thrombocytopenia, various bone manifestations, interstitial lung disease, with atherogenic profile, and cardiac disease secondary to dyslipidemia. Like Type I GD, the onset of Type B ASMD ranges from childhood/adolescence to adulthood, with a wide spectrum of clinical manifestations and disease progression. Recent data confirm that ERT with olipudase alfa (recombinant human acid sphingomyelinase) is safe and effective for the treatment of ASMD Type A/B and B [5, 6]. However, ERT has not been studied yet in the setting of the most severe ASMD Type A, which remains without therapy similar to GD Type II.

Thus, the differential diagnosis of ASMD should include GD given the overlap of several clinical manifestations such as liver dysfunction, splenomegaly, thrombocytopenia, bone involvement, and dyslipidemia. Furthermore, diagnosis of these two diseases can often be delayed due to the non-specific phenotypes.

In the last years, the availability of new technologies and new specific therapies led to consider LSDs as natural candidates for newborn screening (NBS). The NBS program aims to identify pre-symptomatic infants with treatable diseases, based on 10 principles defined by the World Health organization in 1968, known as the “Wilson and Jungner classic screening principles” [7], and a revision has been recently proposed for this specific setting in Italy [8]. Despite its clear relevance among modern diagnostic approaches for LSDs, NBS presents several technical and ethical challenges. A nationwide NBS program is expensive, and requires a great technical and logistical effort, which is not always feasible. Furthermore, some authors claim that NBS for LSDs has some drawbacks such as the diagnosis of asymptomatic newborns who may experience the disease after years with the perspective of a timeless follow up and unpredictable psychosocial impact and costs on children and families [9–12]. Therefore, in the absence of a more effective method, the use of an algorithm to screen a selected population can be a viable alternative to identify the patient at the onset of symptoms, or when the diagnosis is delayed due to the lack of medical awareness, in the effort to prevent irreversible organ damage [13]. The first algorithm for early diagnosis of GD in adult patients was published by Mistry in 2011[14]. In 2014 an algorithm for the diagnosis of GD in children was published by Di Rocco, et al. [15], leading the way for a project on GD screening in a pediatric population at risk, that identified GD in around 10% of enrolled patients [16]. The project had also an educational role, increasing pediatricians’ awareness of this treatable rare disease. The possible overlapping of clinical presentation in late-onset forms of GD and ASMD and the availability of a safe and effective ERT for both diseases led us to implement a new algorithm for the differential diagnosis of these pathological entities. Since this flowchart starts from specific clinical signs such as splenomegaly and hepatomegaly, it is mainly addressed to specialists who are most likely consulted for the evaluation of these signs such as pediatricians, pediatric haematologist and pediatric hepatologists.

## Methods

### Consensus process

The panel of Italian experts included pediatric metabolists and physicians involved in the diagnosis and treatment of LSD. The group used a modified Delphi technique to reach a consensus [15].

Based on their clinical experience and data from published reports, the panel members outlined the general structure of the algorithm and core information flow. As for the previous GD algorithm, a clinical rationale was identified to increase the chance to be applied in clinical practice. All comments and suggestions were reviewed, discussed, and, if accepted, finally implemented. The result is a consensus algorithm for the early diagnosis of both GD and ASMD in a selected pediatric population with organomegaly and cytopenia.

In our opinion, the screening in a selected population needs to meet some criteria: (1) the availability of a safe and effective therapy, (2) the identification of the most frequent symptoms/signs of onset, (3) the development of an algorithm to guide the diagnosis, (4) a specific and sensitive diagnostic method, (5) the identification of the pediatric specialist the patient is more likely to refer to.

### Rationale

The possible diagnostic flowchart was derived from clinical observation of patients with a clinical phenotype compatible with GD later diagnosed with ASMD. Visceromegaly (hepatomegaly or splenomegaly) was chosen as the starting point of the algorithm, being the most frequent sign of onset for both GS and ASMD. Then, the authors identified two parallel diagnostic pathways for GD and ASMD which ultimately end with the Dried Blood Spot Test (DBS).

For the rationale behind the signs and laboratory criteria of the GD side of the algorithm, we refer to the previous paper [15]. Some criteria were revised: (a) eye movement disorders (strabismus and/or oculomotor apraxia) were excluded because only suggestive of later involvement of GD type 3, which initially often manifests only visceral involvement. (b) Tartrate Resistant Acid Phosphatase (TRAP) was also excluded because obsolete for most laboratories.

Similar to GD, the ASMD algorithm focus on specific clinical and laboratory criteria suggesting prompt biochemical testing. The rationale of ASMD algorithm signs and laboratory criteria is reported below:

1) *Increased transaminases*. McGovern et al. reported increased ALT and AST in a cohort of pediatric and adult patients (30 pediatric patients, median age 12 years) with mean alanine transferase values of 65.5 U/L [17]. In a 10-year longitudinal study including 20 pediatric patients and 9 adults, at the initial evaluation, 75% of patients had mildly increased ALT and 65% mildly increased AST [18]. In another study including 30 patients, Hollak CEM et al. reported that 19 of 21 ASMD type B patients had hepatomegaly associated with elevated liver enzymes, especially in younger patients [19].

2) *Dyslipidemia*. It is recognized as a frequent biochemical abnormality in pediatric ASMD. In the prospective study reported above, at the first visit total cholesterol and triglycerides were elevated at baseline in the pediatric group (mean values of 5.71 mmol/L and 2.28 mmol/L respectively) [17]. In a study involving 40 pediatric patients (30 with ASMD type B), hyperlipidemia was characterized by hypertriglyceridemia and increased LDL-C in 62% and 67% of patients respectively. Although 100% of patients showed decreased HDL-C, 13 patients had low HDL-C levels in the absence of hypertriglyceridemia [20]. Coronary artery calcium scores were positive (> 1.0) in 10 of 18 type B patients studied, suggesting that dyslipidemia is associated with early atherosclerotic cardiac disease [20].

3) *Growth retardation*. This non-specific sign is frequently reported in children and adolescents with ASMD type B. Moreover, it is often associated with delayed skeletal maturation and delayed puberty. Wasserstein et al. reported that the average Z-score for height and weight was − 1.24 (29th percentile) and − 0.75 (34th percentile) respectively in a cohort of 23 children and adolescents. 39% (9 out of 23) of those patients had a height below the 5th percentile for age and sex. Thus, the authors postulate that the association between growth retardation and the degree of organomegaly was suggestive of causal relation in ASMD, as well as in type I GD, and ERT is effective in reducing organomegaly and normalizing linear growth. Another study involving 30 pediatric patients reported height Z-scores ranging from − 4.88 to 2.14 (mean − 1.3 ± 1.51), with 29% of patients having height Z-scores < − 2. Growth retardation was particularly prominent in adolescents (mean height Z score − 2.71) [21].

4) *Respiratory involvement*. Few studies concerning respiratory involvement in children with ASMD are available. Guillemot et al. reported on 13 children (1 with type A, 10 with type B, and 2 with type AB). 2 patients with Type B ASMD had bronchitis or bronchiolitis at disease presentation, while 3 patients had asthma. All patients showed radiological evidence of interstitial lung disease [22]. McGovern et al. also reported that 20% of patients had generic respiratory symptoms at the presentation in a cohort of 30 children with ASMD [20]. In the experience of the expert panel, patients asymptomatic for respiratory involvement at disease onset show abnormal diffusing capacity of the lungs for carbon monoxide (DLCO) test or radiological data consistent with interstitial lung disease if tested after ASMD differential diagnosis is postulated. Therefore, we included in the ASMD algorithm the radiological evidence of interstitial lung disease (if available) rather than clinical respiratory involvement, as well as radiological evidence of Erlenmeyer flask deformity in the GD algorithm (if available).

### Diagnostic method

Enzyme activity assessed on DBS by multiplexed tandem mass spectrometry (MS/MS) coupled to specific biomarkers as second-tier analysis, allows for better identification of patients and reduction of false positives.

#### Determination of acid β-glucocerebrosidase [ABG] enzyme activity in DBS

ABG enzyme activity is determined simultaneously with acid α-glucosidase (Pompe disease), β-glucosidase (GD), and α-L-iduronidase (mucopolysaccharidosis type I) activities in a single 3.2 mm DBS punch, using a multiplex MS/MS assay, as previously described [23]. The kit adopted contains substrates and internal standard for acid sphingomyelinase (ASMD) activity too. Assay results were obtained within the 24-h period due to the overnight incubation period. Enzyme activity was expressed as micromoles of substrate hydrolyzed per hour of incubation per liter of blood (umol/l/h). Based on a pre-pilot study on 3500 residual and known patient DBSs, a cutoff of 3 umol/l/h and 1,2 umol/l/h have been established respectively for ABG activity and NB A/B activity, according to 0.2 multiple of median (MOM).

#### Determination of LysoGb1 and Lyso SM in DBS

- Gaucher disease: Lyso-GL-1, the deacylated form of glucosylceramide (GL-1), the major substrate that accumulates in Gaucher disease, is highly specific to Gaucher and in the direct causal pathway of the disease DBSs with low ABG activity should be tested for lysoGb1 and by an LC-MS/MS method, as previously described [24].
- ASMD (formerly Niemann Pick type A/B): DBSs with low sphingomyelinase activity should be tested for lysoSM and by a LC-MS/MS method, as previously described [24].

Recent studies show that these biomarkers (Lyso GB-1 and Lyso-SPM) are useful for diagnosis, decrease with treatment, and their levels correlate with disease severity [25, 26].

## Results

**Fig. 1:**
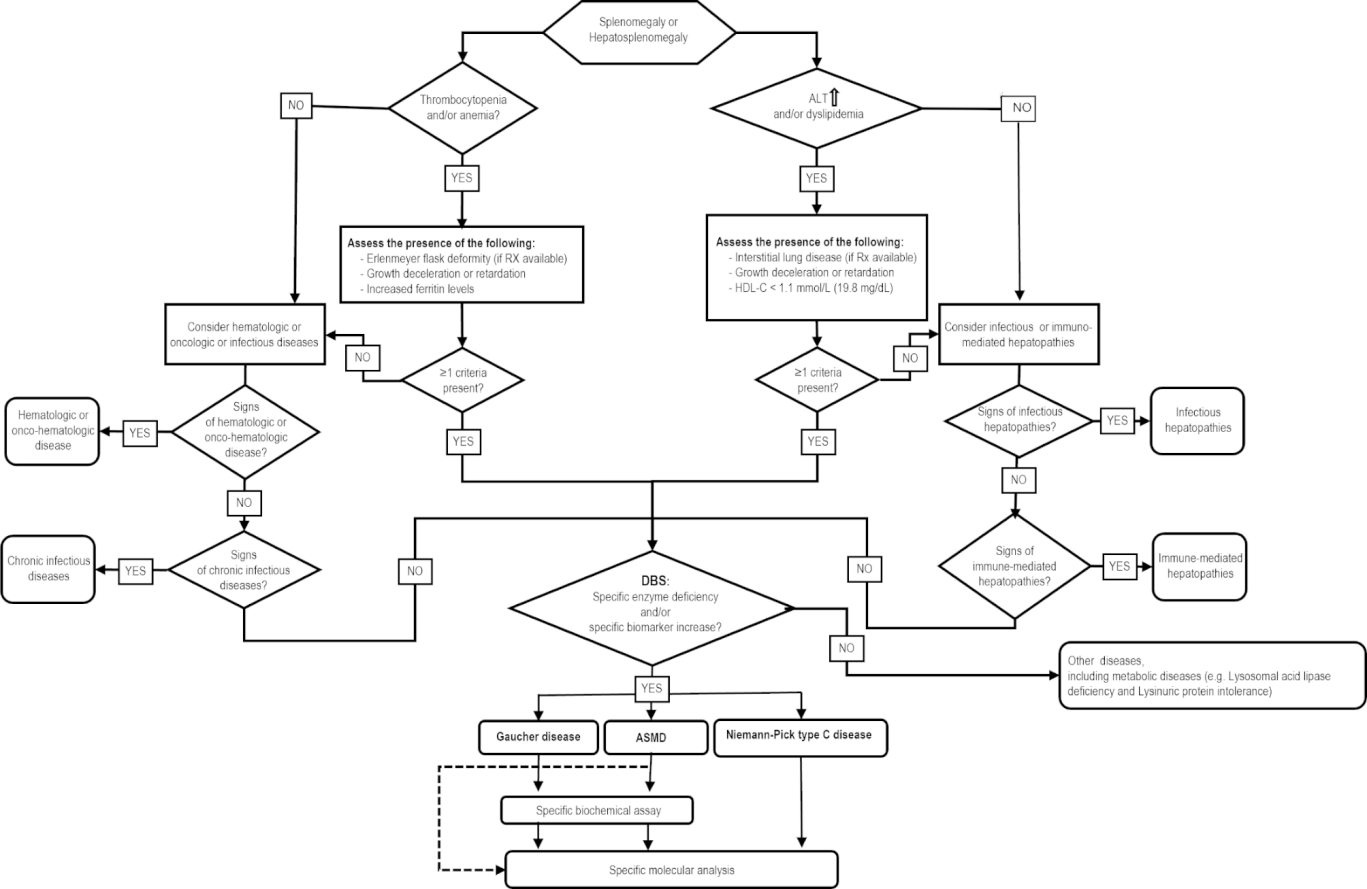
The combined algorithm for GD and ASMD.

The combined algorithm for GD and ASMD is reported in Fig. 1. Pediatric patients with splenomegaly or hepatomegaly are eligible for further diagnostic evaluations.

Patients with Thrombocytopenia and/or Anemia will be assessed for the presence of at least 1 criterion among Erlenmeyer flask deformities, growth deceleration or retardation, increased ferritin levels. If ≥ 1 criteria are met, the patient is eligible for DBS screening. If none of the above criteria is present, the physician should rule out other differential diagnosis (such as hematologic or onco-hematologic disorders, and infectious diseases).

Patients with increased alanine aminotransferase (ALT) levels and/or dyslipidemia will be assessd for the presence of at least 1 criterion among interstitial lung diseases, growth deceleration or retardation, and HDL-C < 1.1mmol/L (19.8 mg/dl). If ≥ 1 criteria are present, the patient is eligible for DBS screening. If none of the above criteria are met, the physician should rule out other differential diagnosis (such as infectious or immunomediated hepatopathies).

Determination of acid β-glucocerebrosidase [ABG] enzyme activity coupled with the determination of LysoGb1 and Lyso-SM will allow for better identification of patients and reduction of false positives for Gaucher Disease and ASMD, eventually leading to the diagnostic confirmation with specific molecular analysis.

## Discussion

We propose a model for the screening of two lysosomal storage diseases (GD and ASMD) in a selected population of pediatric subjects at risk due to the presence of organomegaly and cytopenia (thrombocytopenia and anemia). Both diseases show untreatable rapid neurodegeneration in the infant forms, with death occurring in the first years of life. Nevertheless, the onset of the other forms ranges from childhood/adolescence to adulthood for both diseases. According to the expert panel, these data do not support the utilization of newborn screening for these conditions, but a selected population screening on early presentation is considered more adequate. The measurement of lysosomal enzymatic activities coupled with the measurement of specific biomarkers in DBS is a specific and sensitive diagnostic method.

Based on literature data and clinical experience, it was possible to identify early clinical signs or laboratory abnormalities to draw a combined algorithm to aid GD and ASMD diagnosis. Using this tool, the physician could be able to screen GD and ASMD in a population of patients with splenomegaly or hepatosplenomegaly. Moreover, the algorithm is a good educational tool to enhance the knowledge of these lysosomal diseases.

Another lysosomal disease (Niemann Pick type C) could be suspected at the end of our algorithm. This disease, due to the defect of cholesterol transport, is an untreatable neurodegenerative disease with associated visceral manifestations in infancy and pediatric age [27–29]. The diagnosis, suggested by normal sphingomyelinase and increased lyso-sphingomyelin and confirmed by demonstration of biallelic pathogenetic variants of NPC1 or NPC2, allows early genetic counseling to the family.

Moreover, our algorithm could be used as a viable tool in the differential diagnosis of another treatable lysosomal disease (Cholesteryl Ester Storage Disease, CESD) due to acid lipase deficiency [30]. However, acid lipase cannot be measured with the same DBS method of GD and ASMD [31], therefore we suggest considering CESD and other metabolic diseases after the exclusion of GD and ASMD.

The main limitation of this study is the fact that although this algorithm is drawn from the vast experience of an expert panel, it has not been validated in clinical practice yet. However, the authors worked and expanded upon the previous successful Italian experience of the algorithm for Gaucher Disease proposed by Di Rocco et al. [16], in the effort to streamline the differential diagnosis of GD and ASMD to reduce diagnostic delays. A prospective evaluation of the diagnostic power of this algorithm will be implemented in Italy.

## Conclusions

In conclusion, we propose an algorithm for the screening of a selected population for ASMD and GD, since those entities are not currently among the diseases subjected to NBS in Italy. We believe that this tool will aid pediatricians and other specialists often confronting the differential diagnosis of those rare conditions.

## Data Availability

Not Applicable.

## Abbreviations

LSD: Lysosomal Storage Diseases
GD: Gaucher Disease
ERT: Enzyme Replacement Therapy
SRT: Substrate Reduction Therapy
ASMD: Acid Sphingomyelinase Deficiency
NBS: Newborn Screening
DBS: Dried Blood Spot Test
TRAP: Tartrate Resistant Acid Phosphatase
ALT: Alanine Aminotransferase
AST: Aspartate Aminotransferase
HDL: High Density Lipoprotein
DLCO: Diffusing Capacity for Carbon Monoxide
MS/MS: Multiplexed Tandem Mass Spectrometry
ABG: acid β-glucocerebrosidase
MOM: multiple of median
CESD: Cholesteryl Ester Storage Disease
